# Noninvasive respiratory assistance as aid for respiratory care in neuromuscular disorders

**DOI:** 10.3389/fresc.2023.1152043

**Published:** 2023-05-18

**Authors:** Andrew Graustein, Hugo Carmona, Joshua O. Benditt

**Affiliations:** ^1^ Division of Pulmonary, Critical Care and Sleep Medicine, United States Department of Veterans Affairs, VA Puget Sound Health Care System, Veterans Health Administration, Seattle, WA, United States; ^2^ Division of Pulmonary, Critical Care and Sleep Medicine, Department of Medicine, School of Medicine, University of Washington, Seattle, WA, United States

**Keywords:** noninvasive ventilation, mechanically assisted cough, neuromuscular disease, rehabilitation, chronic respiratory failure

## Abstract

Chronic respiratory failure is a common complication of neuromuscular disease. The use of noninvasive ventilation and mechanically assisted cough can reduce symptoms of hypoventilation, slow lung function decline, improve sleep quality, and in some cases prolong survival in patients with neuromuscular disease. In this article, we review indications for the initiation of noninvasive ventilation and mechanically assisted cough as well as provide recommendations for settings and titration. We discuss the evidence supporting the use of noninvasive ventilation as an adjunct to rehabilitation in patients with neuromuscular disease. Lastly, we review the ethical considerations that are relevant to decisions regarding initiation and cessation of noninvasive ventilation. While noninvasive ventilation and mechanically assisted cough have become standards of care in many forms of neuromuscular disease, most current recommendations are based on expert opinion rather than much-needed data from prospective clinical trials and we emphasize topics requiring future research.

## Introduction

Chronic respiratory failure is a common complication of many forms of neuromuscular disease (NMD). Sequelae of a variety of neuromuscular diseases (NMDs) include weakness in inspiratory, expiratory, and bulbar muscles. While the lung parenchyma may be normal, respiratory muscle weakness can result in chronic hypoventilation leading to hypercarbia, hypoxemia, and sleep disordered breathing (1, 2). The inability to generate a sufficient cough to clear secretions increases the risk of atelectasis, respiratory tract infection, and respiratory failure (3–5).

Patients with chronic respiratory failure due to NMD can benefit from mechanical devices that provide ventilatory support and cough augmentation. The use of noninvasive ventilation (NIV), which is most commonly noninvasive positive pressure ventilation (NPPV) delivered *via* mask or mouthpiece interface, can help to mitigate sleep disordered breathing as well as provide ventilatory support in patients with chronic hypoventilation. Mechanically assisted cough (MAC), either with a simple bag mask and one-way flow valve or mechanical insufflator-exsufflator, can improve secretion clearance and provide lung volume recruitment. The use of NIV and airway clearance therapy are considered standard practice for patients with respiratory failure due to NMD (6).

In this review, we discuss the use and potential benefits of NIV and MAC in patients with chronic respiratory failure due to NMD. We address criteria and timing for initiation in different forms of NMD and describe options for tailoring the ventilatory mode based on disease characteristics. We review NIV in pulmonary rehabilitation and exercise. Lastly, we discuss ethical considerations for initiation and cessation of NIV in patients with NMD.

## Criteria and timing for initiation of respiratory assistance in NMD

In adults, NMDs are due either to adult-onset diseases such as amyotrophic lateral sclerosis (ALS) and spinal cord injury or childhood-onset diseases such as Duchenne muscular dystrophy (DMD) and cerebral palsy where survival into adulthood is increasingly common (7). The most common NMDs in childhood are DMD, spinal muscular atrophy, and congenital muscular disorders including a large group of muscular dystrophies. In adults, ALS, myotonic dystrophy, and limb-girdle muscular dystrophy are the most common NMDs (8). When considering criteria and timing for initiation of respiratory support devices, it is useful to differentiate between slowly progressive and rapidly progressive disease (Table 1) (8). Goals of NIV include the improvement of symptoms of hypoventilation, sleep quality, and overall quality of life. In some instances the use of NIV in patients with NMD may prolong survival. Mechanical assisted cough (MAC) can reduce the risk of pneumonia and atelectasis in patients with NMD and may also prolong survival (4).

**Table 1:** Rates of progression of select NMDs.

Slow (>15 years)	Intermediate (5–15 years)	Rapid (0–3 years)
SMA type 2	DMD	SMA type 1
SMA type 3	Myotonic dystrophy	ALS
Acid maltase deficiency	LGMD	Traumatic SCI

SMA, spinal muscular atrophy; DMD, Duchenne muscular dystrophy; LGMD, limb-girdle muscular dystrophy; ALS, amyotrophic lateral sclerosis; SCI, spinal cord injury. (Adapted from Pinto et al (8).

### Criteria and timing for initiation

The initiation of NIV is based on a combination of patient symptoms and objective measures using pulmonary function testing, arterial blood gas measurements, and polysomnography or desaturation studies (9). Expert opinion drives most recommendations since prospective and randomized trials studying optimal timepoints for initiation are lacking (10). A summary of recommendations from various organizations is described by Hilbert (11). In the United States, a respiratory assist device for NIV may be limited by the Centers for Medicare and Medicaid Services (CMS) reimbursement criteria (12):

(1) Documentation of a restrictive thoracic disorder.
(2) One of the following:
  a. PaCO_2_ on awake arterial blood gas ≥ 45 mmHg while breathing the prescribed fraction of oxygen.
  b. Sleep oximetry with SpO_2_ ≤ 88% for five minutes or more while breathing the prescribed fraction of oxygen.
  c. Either a maximal inspiratory pressure (MIP) < 60 cmH_2_O or forced vital capacity (FVC) < 50% predicted.
(3) COPD is not a significant contributor to the pulmonary limitation.

Recommendations from most other organizations include both objective measures and symptoms of hypoventilation (11). In a survey conducted by the European Respiratory Society in 2019, the primary reason to initiate home NIV was diurnal hypercapnia while quality of life and sleep were the most important goals to achieve (13). In a survey of ALS specialists in the United States, close to 80% of respondents said that they would alter their prescribing patterns for NIV if reimbursement costs were not an issue (9).

MAC can be utilized to generate an augmented cough for patients with ineffective cough (14). MAC is provided using mechanical insufflation, exsufflation, or most commonly both (mechanical insufflation-exsufflation, known as MI-E). A peak cough flow (PCF) of 270 L/min or less is usually the point at which cough assistance is recommended, as patients with peak cough flows below this level are more likely to develop respiratory failure during upper respiratory tract infection (4, 15). Maximal expiratory pressure (MEP) is a component of some guidelines, with a MEP < 60 cmH_2_O being the cutoff for initiating MAC (16). The use of MAC offers the advantage of being applicable in almost all situations and disease states, as it does not require breath coordination from the patient for efficacy, unlike manual lung hyperinflation techniques with resuscitation bags and/or abdominal thrusts. MI-E, however, does add increased cost and care complexity for caregivers.

Below we provide disease-specific recommendations regarding initiation criteria for NIV and evidence supporting NIV use for three of the common etiologies of chronic respiratory failure in NMD: ALS, DMD, and spinal cord injury.

### Amyotrophic lateral sclerosis

ALS is one of the best-studied NMDs yet data to guide NIV initiation and modality is limited. The European Federation of Neurological Societies guidelines recommend initiation of NIV if any one of the following is present (17):

(1) One respiratory clinical symptom related to muscle weakness
(2) FVC < 80%
(3) MIP < −60 cmH_2_O
(4) Sniff nasal pressure (SNP) < 40 cmH_2_O
(5) Significant nocturnal desaturation
(6) PaCO_2_ > 45 mmHg on a morning arterial blood gas.

The SNP allows for testing of patients with bulbar dysfunction who may not otherwise be able to form a mouth seal for other maneuvers and has prognostic value, as does FVC (18).

Noninvasive ventilatory support for individuals with ALS is now standard of practice. The quality of evidence supporting this is not large and there has only been one true randomized controlled trial (RCT) for NIV in ALS (19). An early retrospective analysis suggested that 4 h a day of NIV, usually initiated during sleep, was beneficial (20) while a more recent retrospective analysis shows a potential benefit of NIV use for at least 8 h a day (21).

### Duchenne muscular dystrophy

Treatment with NIV appears to improve quality of life and reduce morbidity and mortality in patients with DMD (4). An American Thoracic Society (ATS) consensus statement recommends sleep laboratory evaluation and NIV titration to normalize ventilation and nighttime oxygen saturation (16) as patients are at an increased risk for sleep disordered breathing. A DMD care considerations working group recommends nocturnal ventilation with any of the following (22):

(1) Signs or symptoms of hypoventilation
(2) Baseline SpO_2_ < 95% and/or end-tidal PaCO_2_ > 45 mmHg while awake
(3) Apnea-hypopnia index > 10/hour on polysomnography OR four or more episodes of SpO_2_ < 92% OR drops in SpO_2_ of at least 4% per hour of sleep.

The same working group recommends daytime NIV for abnormal deglutition due to dyspnea, inability to speak a full sentence without breathlessness, or symptoms of hypoventilation with baseline SpO_2_ < 95% and/or blood or end-tidal PaCO_2_ > 45 mmHg while awake (22). The use of mouthpiece ventilation, in particular, is recommended as a daytime interface option for patients with DMD (10, 16, 22). A variety of cough augmentation techniques in addition to MAC including glossopharyngeal breathing and expiratory augmentation with abdominal thrusts are described elsewhere (4). The use of noninvasive ventilation in DMD has clearly extended life expectancy although no long term randomized trials have been performed (23, 24).

### Spinal cord injury

The diaphragm is the principal muscle of inspiration and is innervated by the cervical nerve roots C3-C5. The principal muscles of expiration (and therefore cough) are the internal intercostals, innervated by the corresponding thoracic nerves, and the abdominal muscles, innervated by T5-L1 (25). Accessory muscles of inspiration and exhalation receive innervation from most of the spinal cord.

In a traumatic spinal cord injury, ventilatory impairment depends on the involved spinal cord level and the completeness of the injury (25). Mechanical ventilation is usually required following injury to C3-C5 (26), though most patients with a lesion at C4 or below can be weaned (27). Injuries to C6-C8 compromise the ability to generate forced exhalation and cough. For thoracic spine injuries, cough effectiveness improves as the level of injury decreases (25). The use of chest physiotherapy including MAC can be critical in patients with spinal cord injuries as cough function is almost invariably abnormal in these individuals. Reviews providing further details of chest physiotherapy techniques can be found with Schilero (25) and Mansel (27). Sleep disordered breathing is more prevalent in individuals with spinal cord injury; formal sleep study is recommended with complete tetraplegia and should be considered in patients with incomplete tetraplegia and paraplegia (28).

## Role of noninvasive ventilation in pulmonary rehabilitation

Noninvasive ventilation in pulmonary rehabilitation for chronic respiratory failure has been best studied in chronic obstructive pulmonary disease (COPD) rather than NMD. Exercise training can improve exercise tolerance and quality of life in patients with COPD (29, 30). NIV used as an adjunct to exercise-based pulmonary rehabilitation can augment the effects of an exercise program and is most beneficial with severe disease (31).

Regarding pulmonary rehabilitation for NMD there is a lack of evidence due to the heterogeneous nature and varied symptoms and limitations of this class of diseases (31). A Cochrane Review evaluated strength training and aerobic exercise in a variety of muscle diseases, concluding that evidence for benefits of strength training and aerobic exercise remain uncertain (32). The impact of exercise training in patients with ALS is controversial. An early study found a hastening in motor function decline and death in an ALS mouse model exposed to high-intensity exercise training (33). A meta-analysis of exercise training found significant improvements in functional ability and quality of life, noting the poor overall quality of included trials (34).

Benefits of NIV for pulmonary rehabilitation and exercise training in NMD are inconclusive. A prospective trial evaluated the impact of mouthpiece noninvasive ventilatory support on 6 minute walk test (6MWT) outcomes in patients with restrictive disorders (11 kyphoscoliosis, 3 congenital myopathy, and 4 other). The authors found significant improvements in oxygen saturation, median distance walked, and an increase in total ambulation time (35). In ALS, the use of NIV during an exercise program led to a significant improvement in functional independent mobility scale and a slower clinical course relative to controls who were not exercised (36). A Cochrane Review of exercise in ALS from 2013 suggested improvement in ALS functional rating scale favoring exercise but no significant differences in quality of life, fatigue, or muscle strength (37). The use of NIV *via* nasal mask significantly improved exercise endurance time and deceased breathlessness in a study of 7 patients with restrictive ventilatory deficits due to pulmonary tuberculosis sequelae (38).

Other studies have been less promising. A randomized control trial of NIV with chronic respiratory failure due to either COPD or restrictive thoracic disease (kyphoscoliosis or tuberculosis sequelae) found improvements in 6MWT distance due to participation in an exercise regimen that was not impacted by the use of supplemental NIV (39). While NIV did increase endurance time this was primarily driven by the COPD group. In an earlier study of NIV in patients with severe scoliosis already using nocturnal NIV, the addition of a mouthpiece alone or mouthpiece plus ventilatory support incrementally worsened walk distance (40). Potential limitations included the addition of monitoring equipment that may have impaired performance and delays in ventilator triggering on inspiration.

Respiratory muscle training (RMT) can including either inspiratory (IMT) or expiratory (EMT) muscle training. Studies evaluating the benefits of RMT in patients with NMD are scarce and heterogeneity in patient population and trial design limit conclusions. A 2019 Cochrane review of RMT in children and adults with NMD included three trials with ALS, six with DMD, and two with other NMDs (41). None provided long term data and most did not include information about adverse events. The authors concluded that RMT may improve lung capacity and muscle strength in some NMDs, though these improvements may not lead to clinically meaningful effects on quality of life or physical functioning (41). A recent meta analysis representing a variety of NMDs suggested improvements in lung volume and respiratory muscle strength, noting substantial study heterogeneity limiting confidence (42). A Cochrane review of RMT in patients with cervical spinal cord injury found significant improvements in vital capacituy, MIP, and MEP with the use of RMT (43). The authors noted the need for further studies to identify impacts of RMT on functional outcomes such as dyspnea and quality of life.

To summarize, most available evidence suggests a lack of harm with exercise in NMD, with supplemental NIV to support exercise either beneficial or at least non-harmful. Most trials suggest a lack of harm with the use of IMT or EMT and potential short-term improvements in strength and pulmonary function testing but have not evaluated longer term clinical outcomes. High-quality, robust research is desperately needed.

## Ventilatory mode of noninvasive ventilation

The use of various NIV modes of ventilation are varied and depend on device accessibility, patient and clinician preference, and local experience (13).

### Mode of ventilation

The main modes of NIV that are used to support patients with NMD include bilevel pressure support in either a spontaneous (S) or spontaneous/timed (S/T) mode or volume-assured pressure support (VAPS) mode (44). Among a survey of European clinicians who care for patients with NMD, S/T was the most frequent mode of ventilation used (13). Mouthpiece ventilation is performed with volume-control ventilation. Continuous positive-airway pressure (CPAP) is not adequate to treat hypoventilation that occurs with NMD.

S/T mode is preferred over S mode since it includes a back-up rate in case of ongoing bradypnea (44) or apnea. Initial parameters include an expiratory positive-airway pressure (EPAP) of 5 cmH_2_O and Inspiratory positive-airway pressure (IPAP) of 5–10 cmH_2_O above the EPAP. The backup respiratory rate is set below the patient's intrinsic rate with the goal that they trigger the majority of their own breaths. The clinician should then monitor for a resulting tidal volume and minute ventilation that is appropriate, generally 6–8 ml/kg of ideal-body weight (45). VAPS offers the theoretical advantage of titrating the pressure support levels to achieve a targeted tidal volume and minute ventilation and has been used with success in ALS (46).

### Interface

Interface options include nasal-only masks, nasal and oral hybrid masks, and full face masks. Mask interfaces are often paired with a pressure-cycled mode of ventilation such as S/T as it allows for both an IPAP and EPAP setting to be maintained. Mouthpiece ventilation uses a plastic mouthpiece that the patient places to their lips to receive a prescribed tidal volume and allows more freedom and comfort compared to a nasal or face mask. With mouthpiece ventilation, a volume-cycled mode of ventilation can be used, as the patient will have a purposeful leak when they exhale to the room, as opposed to back through a limb of the ventilator circuit (6).

### MAC techniques

MI-E functions by providing alternating inspiratory and expiratory pressures for a set duration *via* a face mask or tracheostomy tube (47) to stimulate cough and mobilize secretions. Patients should be introduced to MI-E early in their disease course, while they are healthier, in order to familiarize caregivers with the device before the patient has an upper or lower respiratory illness that increases secretions (48, 49). Regarding settings, shorter, lower inspiratory pressure phases paired with longer, more negative pressure expiratory phases have been shown to be effective and well-tolerated (49) with minimal complications.

Lung Volume Recruitment (LVR) is a manual technique using a resuscitator bag that can augment cough by augmenting lung elastic recoil at higher volumes. LVR may slow loss of pulmonary function over time (50). It is less expensive than MI-E but generally requires an assistant to perform.

### Management of salivation

Siallorrhea can present a challenge to patients with bulbar muscle weakness. Such patients do not produce more saliva than normal; rather, they are not able to effectively manage the same secretion volume (51, 52). For patients with NMD and sialorrhea, the American College of Chest Physicians (ACCP) suggest a therapeutic trial of an anticholinergic agent as first-line therapy. For patients with inadequate response or side effect intolerance, the ACCP suggests either botulinum toxin injection to the salivary glands or salivary gland radiation therapy (53). A more comprehensive description of disease-specific patterns of dysphagia and options for management of sialorrhea can be found in Britton et al. (51).

### Monitoring and follow up

Patients with NMD are typically seen for routine clinic visits every 3–6 months. Regular monitoring of spirometry, MIP/MEP, and peak cough flow is recommended. The ACCP suggests pulmonary function testing at least every 6 months with further testing intervals determined by the patient's specific rate of disease progression (53). Some form of regular CO_2_ monitoring is also performed. Portable or in-office end-tidal or transcutaneous CO_2_ monitoring permit the foregoing of an arterial blood gas (54, 55). There is no data that strongly supports one modality of monitoring over another (56). Polysomnography is not routinely recommended in adults, though for symptomatic patients with normal pulmonary function testing and overnight oximetry, polysomnography can determine whether NIV is clinically indicated (53).

After initiation of NIV, patients should be monitored for evidence of improvement. This includes checking mask fit, reviewing the device data download, and following clinical efficacy. Patients who previously had poor sleep with morning fatigue and headache, daytime somnolence and lower energy should report improvement in these symptoms (10).

## NIV failure and ethical dilemmas

Many of the ethical considerations around NIV use center around the decisions to initiate and discontinue NIV, as well as considerations around the high resource utilization involved. When considering NIV support for chronic respiratory insufficiency in NMD, a discussion around prognosis, life expectancy, and evaluation of patient decision-making are necessary. This requires navigating a multitude of ethical issues, including decision making in light of possible prognostic uncertainty, evaluating and respecting patient and/or surrogate choices, as well as advance care planning (57, 58). These issues are particularly challenging where the trajectory may be uncertain or for diseases that are progressive and ever-evolving.

NIV offers the ability to extend life expectancy in diseases that may progressively reduce patients' quality of life and independence, such as ALS. Only patients can determine what constitutes an acceptable quality of life and what treatments are worthy or too burdensome. Using the principles of patient autonomy and beneficence, healthcare providers should provide honest information in a caring manner to help patients and their caregivers navigate the decisions around initiation and discontinuation of NIV (59). Clinicians should be aware of their own biases (60) and feelings around the perceived value of certain treatments (61), as they may encounter patients who choose diametric paths when facing similar scenarios and should attempt to counsel both with equal respect and compassion (60). Similarly, there may be disagreement among caregivers and family members, with the need for the clinician to likewise help families reconcile these differences.

At some point NIV may fail to provide appropriate respiratory support (e.g., progressive bulbar symptoms) or their quality of life may no longer be acceptable regardless of the respiratory support they are on. Patients and care givers require support to make well-informed decisions if they choose to discontinue life-support therapy such as NIV (or invasive mechanical ventilation) if patients determine they no longer have an acceptable quality of life (62–64). Advanced planning and the introduction of palliative care services before these issues arise can help avoid conflict and distress (65). This allows time to build a trusted patient-clinician relationship as well as the ability to generate an appropriate hospice plan for those that choose not to continue respiratory support (62).

With high resource utilization, equitable access to healthcare resources for these patients is an issue (66, 67). Even in high resource settings, health and income inequality (e.g., health insurance coverage variability), and local environmental factors such as availability of qualified healthcare professionals (68) may limit access to care. During the COVID-19 pandemic, regions with high levels of hospitalizations due to ill patients have had to contend with resource allocation issues. Healthcare rationing and triaging proposals could target those with perceived less value to society, such as those with neuromuscular disease (68–70). However, ethical frameworks for resource allocation are clear that disability alone should not be used as a bias against receiving a scare resource, and instead longevity, life-expectancy, and ability to make meaningful recoveries as part of the ethical consideration for resource allocation (71).

## Conclusion

Noninvasive ventilatory support and MAC have become standards of care for patients with weakness due to NMD. Most recommendations for timing of initiation and initial settings are based on expert opinion since high-quality clinical trials are lacking. Evidence promoting exercise and pulmonary rehabilitation in NMD, as well as the utility of NIV to support these regimens, is promising but similarly limited. It is vital for healthcare providers be aware of the limited evidence that is available to help patients with NMD navigate complex decisions about initiation and cessation of NIV.
